# 

**Published:** 1874-12

**Authors:** 


### TABLE OF CONTENTS.

ORIGINAL COMMUNICATIONS.

Hydrate Chloral. By Alban S. Payne, M. D., of Virginia, - - - 685

Phosphorus Acid—Its Compounds, and their Therapeutic Value in Nervous Diseases. By T. Curtis Smith, M. D., of Middleport, Ohio, - - 688

Successful Treatment of an Opium Habit, Morphia, Hypodermically, of Nearly Seven Years Standing. By J. B. Mattison, M. D., Chester, N. J., - 699

Difficult Parturition. By L. B. Anderson, M. D., of Virginia, - . - 707

SELECTIONS.

Puerperal Insanity. By W. W. Goulding, M. D., of Taunton, Mass., - - 709

The Therapy of Diarrhœa. By F. A. Hartsen, Marseilles, - - - 711

The Abortive Treatment of Gonorrhœa in the Male by Injection of Clay-Earth. By Frederick William Godon, A. B., M. D., - - - - - - 717

REMARKS, GLEANINGS AND EXTRACTS.

Excoriated Nipples—720. Guavana for the Cure of Sick Headache—720. Picric Acid as a Test for Albumen—721. Villate's Mixture and Its Uses—721. Emulsio Carnis—724. Chronic Bright's Disease—724. Simbat on the Use Chloriee of Zinc in the Treatment of Fistula—725. Bromide Potassium—726. Treatment of Acute and Chronic Bronchitis and Asthma—727. Gonorrhæa Mixture—727. Oxide of Zinc in the Diarrhœa of Infants and Young Childreu—728. Emulsion of Cod Liver Oil—728. Chloral in Threatened Miscarriage—729. Burns—729. Administration of Perchloride of Iron—729. Phenic Acid in the Treatment of Cutaneous Affections—720. Tincture Eucalyptus Globulus in Intermittent Fever—730. Facial Erysipelas—730.. Cosmetic Surgery—731. Local Treatment in Pulmonary Cavities—731. Test for Impurities in Water—731. Treatment of Furuncles—732. Treatment of Gonorrhœa—732. Thudicum Douche—732. A NeW Diaphoretic—732, Vaginitis—733. Amyl Hydride—733. Drssing with Magnesia—733 Retention of Urine Relieved by Ice in the Rectum—733. Pueperal Mania; Treatment by Chloral Bromide of Prtassium—734. Delirium Tremens—734. Acute Articular Rheumatism—734. Lumbago—734. Hemorrhoids: The Elastic Ligature—734.

EDITORIAL AND MISCELLANEOUS.

To Our Subscribers, - - - - - - - - - - - 735

To Our Asiistant Editors and Contributors, - - - - - - 735

The Old and the New, - - - - - - - - - - 736

Advertising Department - - - - - - - - - - 738

Cincho-Quinine, - - - - - - - - - - - 738

Artificial Legs, - - - - - - - - - - - - 738

Vacine Virus, - - - - - - - - - - - - 739

Capsnles, - - - - - - - - - - - - 739

Fluid Extract of Gelsenium, - - - - - - - - - 739

Reviews, - - - - - - - - - - - - - 739

